# Pharmacological Screening of *Viola odorata* L*.* for Memory-Enhancing Effect *via* Modulation of Oxidative Stress and Inflammatory Biomarkers

**DOI:** 10.3389/fphar.2021.664832

**Published:** 2021-05-28

**Authors:** Uzma Saleem, Sundas Hira, Fareeha Anwar, Muhammad Ajmal Shah, Samia Bashir, Roua S. Baty, Reem H. Badr, Renald Blundell, Gaber El-Saber Batiha, Bashir Ahmad

**Affiliations:** ^1^ Department of Pharmacology, Faculty of Pharmaceutical Sciences, Government College University, Faisalabad, Pakistan; ^2^ Riphah Institute of Pharmaceutical Sciences, Riphah International University Lahore, Lahore, Pakistan; ^3^ Department of Pharmacognosy, Faculty of Pharmaceutical Sciences, Government College University, Faisalabad, Pakistan; ^4^ Department of Biotechnology, College of Science, Taif University, Taif, Saudi Arabia; ^5^ Department of Plant Physiology Botany and Microbiology, Faculty of Science, Alex University, Alexandria, Egypt; ^6^ American University of Malta, Triq Dom Mintoff, Bormla, Malta; ^7^ Department of Pharmacology and Therapeutics, Faculty of Veterinary Medicine, Damanhour University, Damanhour, Egypt

**Keywords:** *Viola odorata*, memory-enhancing activity, oxidative stress, lipopolysaccharide, cyclooxygenase-2, tumor necrosis factor–alpha

## Abstract

**Purpose:** Alzheimer disease (AD) is a progressive neurodegenerative disorder that is caused by neuroinflammation and oxidative stress. The present study aimed to characterize and then investigate the memory-enhancing potential of *Viola odorata* methanolic extract in lipopolysaccharide (LPS)–treated mice.

**Methods:** *V. odorata* characterization was done by using the GCMS technique. Neuroinflammation was induced by the intracerebroventricular administration of LPS at a dose of 12 µg. Animals were divided randomly into six groups (*n* = 10). Group I was normal control, which was given vehicle. Group II was disease control, which received LPS (12 µg) *via* the intracerebroventricular route. Group III was standard, which was administered with donepezil (3 µg) orally for 21 days. Groups IV–VI were the treatment groups, which were administered with the extract at 100, 200, and 400 mg/kg dose levels orally respectively for 21 days. Groups III–VI received LPS (12 µg) on the first day along with their treatments. During the treatment, the animals were assessed for memory retention by employing different behavioral paradigms namely elevated plus maze, passive avoidance, foot shock and open field. Various mediators [endogenous antioxidants, neurotransmitters, and acetylcholinesterase (AChE)] involved in the pathogenesis of AD were quantified by using the UV spectrophotometric method.

**Results:** Extract-treated groups showed a remarkable improvement in cognitive impairment in all behavioral paradigms. Oxidative stress biomarkers, that is, superoxide dismutase, catalase, and glutathione were raised dose-dependently in the treatment groups with a dose-dependent decrease in the malonaldehyde and AChE levels in the brains of the treated animals. The treatment groups showed decreased levels of inflammatory biomarkers, that is, tumor necrosis factor–alpha, nuclear factor kappa light-chain enhancer of activated β-cells, and cyclo-oxygenase, which supports the therapeutic effectiveness of the treatment.

**Conclusion:** Based on behavioral, oxidative stress biomarker, and neuroinflammatory data, it is concluded that *V. odorata* possesses memory-enhancing activity and may prove a beneficial role in the management of AD.

## Introduction

Alzheimer’s disease (AD) is a progressive neurodegenerative disorder that commonly affects people older than 60 years (Wu et al., 2018). The Lancet communication report on dementia reported that the risk factor for the neuropathological development of dementia is less in early life (<45 years) and increases gradually from mid life (45–65 years) to later life (>65 years). The key indicative biomarkers of AD are amyloid beta (Aβ) and tau proteins. According to the Lancet communication report on AD, these biomarkers never develop in people who have normal cognitive functions (Livingston et al., 2020).

Neuroinflammation, mitochondrial dysfunction, protein misfolding, oxidative stress, genetic disturbance, and aggregation of misleading proteins are the causes of neurodegenerative diseases (Kamil et al., 2019). AD is a devastating disorder that slowly causes degeneration of neurons and impairs cognitive abilities. The brain loses its cell-to-cell connection (Wenk, 2003). The effect of AD on memory capacity is not equal. Initially, short-term memory is affected followed by episodic memory. At the start, there is difficulty to obtain new memories and remembering observed facts. As the disease advances, confusion, aggression, irritability, mood swings, language breakdown, and long-term memory loss may occur (Reitz et al., 2011). Certain regions of the subcortical and cerebral cortex of the brain lose neurons and synapses in AD. This results in the degeneration of the affected areas including the cingulate gyrus, frontal cortex, parietal lobe, temporal lobe, and hippocampus (Kumar and Singh, 2015). Although the cause of AD is yet not clear, some promising reasons have been found. A decrease in the synthesis or increased breakdown of acetylcholine (ACh) neurotransmitter could be the cause of AD. The serotonergic system imparts its effect on memory by regulating the activity of ACh (Kandimalla and Reddy, 2017). The other major theory states that two kinds of proteins, Aβ and Tau proteins, run out of control in AD, which function actively in the healthy brain (Wenk, 2003). Tau proteins nourish the brain by supplying nutrients to the brain nerve cells. This damaged protein entangles itself inside the nerve cell bodies and impedes the nutrient supply to the cell. Hence, the transport system of neurons collapses, which destroys the brain cells effectively (Lee et al., 2005). Aβ protein is responsible for the brain’s normal activity. Deposits or aggregates of Aβ protein are formed in the initial stages of AD, which distorts the communication of neurons by causing neuroinflammation. Aβ proteins are also responsible for the ineffective concentration of dopamine and norepinephrine, leading to cognitive dysfunction (Bitel et al., 2012). Another significant neurodegenerative pathway for AD is oxidative stress which results due to the overproduction of reactive oxygen species (ROS) and gradual loss of antioxidants defense mechanism. Antioxidant therapy has proven to ameliorate cognitive impairments. Plants are the major source of antioxidants; therefore, natural products can be a favorable therapeutic approach for AD (Saxena et al., 2008).

*Viola odorata* Linn. (Sweet violet) is a perennial, short plant that belongs to the family Violaceae that is commonly found in open lands and road edges (Trusov, 2014). The herb is extensively used in Unani and Ayurvedic medicines. Its flowers are edible and consumed as a salad. *V. odorata* has been proved to have a variety of biological activities (Colgrave et al., 2008) namely, anti-inflammatory, antimicrobial, analgesic, anticancer, diuretic, laxative, and antioxidant (Colgrave et al., 2008). Different phytochemical constituents like cyclotides, steroids, flavonoids, alkaloids, vitamins A and C, saponins, and tannins are found in alcoholic and aqueous extracts of aerial parts of the plant (Akhbari et al., 2012). The whole plant has been used as febrifuge and used in infantile disorder, lung troubles, and diaphoretic actions (Ahmad et al., 2009). *V. odorata* is considered effective against various neurodegenerative diseases such as AD, Parkinson’s disease, epilepsy, and anxiety owing to its anti-inflammatory and antioxidant potential (Vishal et al., 2009).

Neuroinflammation can be induced by different chemicals, but the neuroinflammation induction by the lipopolysaccharide (LPS) is a popular method. LPS is a molecule present in the outer membrane of gram-negative bacteria (Schumann et al., 1990). Neuroinflammation is considered to play a significant role in the pathology and clinical manifestations of AD due to the overexpression of inflammatory agents in the affected regions of the brain (Miklossy et al., 2006). LPS, an inflammation activator, has been accounted for Aβ plaques and memory impairment (Lee et al., 2008). LPS has been proved to cause memory impairment by the stimulation of signaling pathway of pro-inflammatory cytokines like tumor necrosis factor–alpha (TNF-α level) and nuclear factor kappa B (NFK-β). The current study aims to characterize and investigate the effect of *V. odorata* on the cognitive functions of mice by employing the behavior models and determining various biochemical markers.

## Materials and Methods

### Plant Collection and Extraction

The plants containing aerial parts were collected from a local nursery in Lahore, Pakistan. The plant samples were authenticated by a botanist and were stored in the herbarium of the Botany Department of the University of Punjab, Lahore, Pakistan, under voucher number LAH#02720. *V. odorata* plant material was dried under shade for two weeks and then ground electrically into a fine coarse powder. Powdered plant material (1000 g) was extracted by using the maceration process with methanol (3,400 ml), and 1400 ml water in the ratio of 2:1 for 7 days at room temperature. After a week, this mixture was filtered, and then by using a rotary evaporator, solvent was evaporated at 60°C under reduced pressure. It was dried in a dried oven, and a dark brown thick paste was obtained. This extract was weighed, and the percentage yield was determined by using the formula given below. Then, it was stored in an airtight jar in a refrigerator at 2–8^°^C for phytochemical and experimental analysis.

$$\text{Percentage Yield }=\frac{\text{Actual yield}}{\text{Mass of powdered material}}\text{*}100.$$

### Quantitative Phytochemical Analysis

#### Total Flavonoid Contents

The total flavonoid contents were determined by using the method of Saleem et al. (2012) with little modifications. The plant extract (0.2 ml) was mixed with 0.1 ml of 10% aluminum nitrate (Al_2_NO_3)_, 0.1 ml of 1 M potassium acetate, and 4.6 ml of distilled water. This mixture was allowed to stand at room temperature and was incubated for 50 min. Rutin was used as standard. Absorbance was determined at 506 nm. A regression line of rutin was used to calculate total flavonoid contents in the sample plant extract (Saeed et al., 2012).

#### Total Phenol Contents

First, 0.5 ml of plant extract (100 µg/ml) was dissolved in 2 ml of the Folin–Ciocalteu phenolic reagent, which was diluted in a ratio of 1:10 with deionized water. This mixture was neutralized by adding a 4 ml solution of sodium carbonate (7.5% w/v) and incubated for 30 min. Absorbance was measured at 765 nm. Gallic acid was used as a reference standard. The total phenolic contents were measured by using the linear equation of gallic acid (Ainsworth and Gillespie, 2007).

### Gas Chromatography–Mass Spectrophotometry (GC-MS) Analysis

GCMS analysis of phytochemical constituents of *V. odorata* was performed by using an Agilent model 7890 B gas chromatography (Agilent Technologies, CA, United States) equipped with a DB-5MS (5% phenyl-polymethyl siloxane) capillary column (3 mm × 0.25 mm, film thickness 0.25 µm) interfaced to the Agilent 5997C mass-selective detector. Initially, the temperature of the oven was held at 100°C for 1 min and then increased at a rate of 10°C/min up to 300°C. Helium gas was used as carrier gas at a flow rate of 1 ml/min. The temperature of the detector and injector was 280 and 250°C, respectively. The mass spectrum was collected at 70 eV ionization voltages over the range of 0–340 m/z. Electron multiplier voltage was set at 1150 V. Percent relative peak was used to investigate the quantity of all identified compounds. The identification of compounds present in VOME was carried out by comparing the known compounds available in the National Institute of Standards and Technology (NIST) library databases (Jasim et al., 2018).

### Experimental Animals

The study was carried out on Swiss mice (male and female) weighing 20–40 g. They were kept in the animal house of Riphah International University, Lahore, Pakistan. The animal house was maintained at standard conditions (12 h light and 12 h dark cycles, 22 ± 3°C temperature and 45–55% humidity) with free access to food and water. The animals were acclimatized to standard conditions 5 days before the start of experimental studies and were caged separately in groups of five per cage.

### Ethical Approval

The experimental protocols were approved by the Ethical Committee of Riphah Institute of Pharmaceutical Sciences, Lahore, Pakistan, with the approval number of REC/RIPS-LHR/039 and conducted according to the National Institutes of Health (NIH) guidelines for care and use of laboratory animals.

### Experimental Design

LPS (vial had 2000 µg which was dissolved in 2000 µl normal saline; 1 µg/µl solution was prepared) solution was administered *via* an intracerebroventricular (I.C.V.) route at a dose of 12 µg (12 µl volume was administered) by using a stereotaxic apparatus on the zeroth day of the study to all groups of animals, except the control group (Wu et al., 2019). Animals were divided randomly into six groups (*n* = 10). Group I was normal control, which was given vehicle (normal saline). Group II was disease control, which received LPS (12 µg) *via* the I.C.V. route. Group III was standard, which was administered with donepezil (3 µg) orally for 21 days. Groups IV–VI were the treatment groups, which were administered with extract at 100, 200, and 400 mg/kg dose levels orally, respectively, for 21 days. All the doses were prepared in normal saline.

All the animals were sacrificed at the end of the study under isoflurane (3–5% isoflurane diluted with oxygen) anesthesia, and the brains were excised from each mouse to perform biochemical analysis on tissue homogenate.

#### Behavioral Tests

##### Morris Water Maze Test

The Morris water maze test (MWM) was used to evaluate spatial learning and memory in experimental animals. It was performed for five consecutive days (i.e., 8th to 12th days of the study) of which training was conducted on the first four days, and the probe test was performed on the 5th day (Wu et al., 2019). The apparatus consisted of a circular pool with a diameter of 150 cm and a height of 50 cm, which was filled with water up to 40 cm. Water temperatures were maintained at 26 ± 1°C. The water maze was divided into four quadrants, that is, east, west, north, and south. A platform was placed at the center of any of the quadrants in the water which was 2 cm above the water level during training sessions, and was kept in the same position during training. The mice were placed in the water facing the wall of the pool. The starting point was changed on each training day (Niquet et al., 2016). The animals were allowed to find the platform for 60 s. However, if an animal failed to find the platform within the acquisition time, it was guided to the platform. This procedure was repeated with each animal for the first four days of training. On the fifth day, a probe test of 60 s was carried out in which water was made opaque with nontoxic food color and the platform was removed. Different parameters like escape latency time (time taken to find the platform), time spent in the platform quadrant, and the number of crossings in the platform quadrant were recorded (Wu et al., 2019). Shorter escape latency time is an indication of successful learning.

##### Elevated Plus Maze Test

The spatial memory of animals was evaluated by using the elevated plus model (EPM) (Hope et al., 2019). It was performed on the 14th and 15th days of the study. The 14th day (day 1) was considered as a training day, whereas retention of memory was examined on the 15th day (day 2) of the study. The EPM apparatus was in plus shape with four arms (two open, 25 × 5 cm and two closed (25 × 5 × 16 cm) with a central platform (5 × 5). The apparatus was elevated 24 cm above the floor (Ari et al., 2019). Transfer latency time (TL) (time taken by an animal to enter from the open arm to any closed arm with all its four paws) was observed by placing each animal at the end of the open arm facing away from the central platform. Each mouse was freely allowed to explore the apparatus for 90 s. If any animal failed to enter into a covered arm within the assigned period (90 s), then it was trained to enter the closed arm. A decrease in transfer latency indicated cognitive improvement (Herbst et al., 2019).

##### Fear Conditioning Test

This test was performed to assess learning and memory associated with fear (Shoji et al., 2014). The test was performed on the 19th, 20th (training), and 21st (assessment) days of the study. The apparatus comprised a square-shaped acrylic chamber having an electric grid floor along with a shock generator and a sound source. A light source was also attached to illuminate the chamber. The sound speaker worked as an auditory cue as a conditioned stimulus (CS), whereas the grids were connected with a shock generator to provide a foot shock as an unconditioned stimulus (US) (Shoji et al., 2014). Each treated mouse was placed in the test apparatus to explore the chamber for 60 s. After this, a musical tone (2.8 kHz) was played as CS for 30 s, and foot shock (0.7 mA) was given as the US on the last 2 s of the tone. The procedure was repeated twice after every 60-s interval (Angelo et al., 2003). On the assessment day, the treated mice were evaluated for the retention of memory by repeating the same procedure without applying foot shocks. Freezing time was observed. Freezing is defined as a lack of movement, except breathing (Ikegami et al., 2000).

##### Passive Avoidance Test

The passive avoidance model was used to evaluate the episodic memory of animals (Kameyama et al., 1986). The test was conducted in three sessions on the 21st (training) and 22nd (assessment) days of the study. Step-down latency (SDL) was measured in this test (Kameyama et al., 1986). The apparatus consisted of a square wooden box (40 × 40 × 50 cm^3^) with an electrifiable floor made up of stainless steel rods that were 1 cm apart from each other. Three walls of the box were made up of wood, whereas the fourth wall was made up of glass. A wooden platform (8 × 5 × 1.5 cm) was placed at the center of the box. The treated animal was delicately placed on the wooden stage, and SDL (the time when an animal steps down the floor with all of its four paws) was observed. When the animal stepped down from the platform and placed all of its paws on the floor, electric shock (0.4 mA) was applied for 3 s. The animals that step down within 2–15 s were chosen for the second session after 90 min of session 1 (Nasehi et al., 2010b). On the 22nd day, the mice that exhibited the SDL less than 60 s were evaluated for memory retention in session 3. Increased SDL indicated improvement in cognitive function (Nasehi et al., 2010a).

### Estimation of Oxidative Stress Biomarkers

#### Preparation of Tissue Homogenate

Animals were anesthetized by using isoflurane (Cartner et al., 2007), and then, they were sacrificed by cervical dislocation to excise their brain. Each separated brain was weighed and rinsed with chilled normal saline. A tissue homogenate (10% w/v) was prepared in 0.03 M phosphate buffer (pH 7.4) with a ratio of 1:10. The homogenate was centrifuged at 3,000 rpm for 15 min. The supernatant layer was utilized to measure biochemical markers.

#### Estimation of Superoxide Dismutase (SOD)

Potassium phosphate buffer (0.1 M, pH 7.4) 2.8 ml and pyrogallol solution 0.1 ml were added to 0.1 ml of brain homogenate. This mixture was used to measure absorbance at 325 nm on a UV spectrophotometer (Bhangale and Acharya, 2016).

#### Measurement of Catalase Activity (CAT)

The reaction mixture was prepared by the addition of 0.05 ml of tissue homogenate, 1 ml of hydrogen peroxide (H_2_O_2_), and 1.95 ml of phosphate buffer (50 mM, pH 7). Absorbance was recorded at 240 nm (Kim et al., 2016). Catalase activity was determined by using the given formula:

$$\text{Catalase activity }=\;\frac{\text{δOD }}{\text{E}\times \text{Vol of sample }\left(\text{ml}\right)\times \text{mg of protein}},$$

where δOD = change in absorbance per minute and E = extinction coefficient of hydrogen peroxide (0.071 mmol cm^−1^). Protein contents were calculated by following Lowery et al.’s (1951) method (Classics Lowry et al., 1951).

#### Measurement of Malondialdehyde (MDA)

One milliliter of brain homogenate was added to 3 ml of thiobarbituric acid (TBA) reagent (which contained 5 N HCl and trichloroacetic acid; TCA) to make a reaction mixture which was heated for 15 min at 90^°^C. After cooling, it was centrifuged at 3,500 g for 10 min. The resultant pink-colored supernatant was used to measure absorbance at 532 nm (Saxena et al., 2008).

The following equation was used to calculate the MDA level:

$$\text{Conc}.\text{of MDA }=\frac{{\text{Abs}}_{532}\times 100\times {\text{V}}_{\text{t}}}{\left(1.56\times 10^{5}\right)\times {\text{W}}_{\text{T}}\times {\text{V}}_{\text{U}}},$$

where Abs_532_ = absorbance, V_T_ = total mixture volume, 1.56 × 10^5^ = molar extinction coefficient, W_T_ = weight of the brain, and V_U_ = aliquot volume.

#### Measurement of Reduced Glutathione (GSH) Level

Brain homogenate, 1 ml, was mixed with 1 ml of 10% trichloroacetic acid, and it was centrifuged at 2000 g for 10 min. The resultant supernatant was separated in which 2 ml of phosphate buffer (pH 8.4) and 0.5 ml of DTNB reagents were added. The absorbance was measured at 412 nm (Saxena et al., 2008).

The following equation was used to measure the GSH level:

$$\text{GSH Level }=\frac{\text{Y}-0.00314\;}{0.0314}\times \frac{{\text{D}}_{\text{F}}}{{\text{B}}_{\text{T}}\times {\text{V}}_{\text{U}}}.$$

Here, Y = absorbance at 412 nm, DF = dilution factor, BT = brain tissue homogenate volume (1 ml), and VU = volume of aliquot (1 ml).

#### Estimation of Acetylcholinesterase Activity

Tissue homogenate, 0.4 ml; 2.6 ml of phosphate buffer; and 100 µl of DTNB were mixed thoroughly, and absorbance was measured at 412 nm using a UV spectrophotometer. After stable reading, it was recorded as basal reading. To this mixture, 20 µl acetylthiocholine iodide was added as a substrate, and change in absorbance was measured up to 10 min with an interval of 2 min (Talpate et al., 2014).

The average change in absorbance was calculated by using the following formula, and the activity of acetylcholinesterase (AChE) was expressed as µM/l/min/gm of tissue.

$$\text{R}=5.74\left(10^{-4}\right)\times \frac{\text{ΔA}}{{\text{C}}_{\text{O}}}.$$

Here, R = rate in moles substrate hydrolyzed per minute per gram of tissue, ΔA = change in absorbance per minute, and C_o_ = original concentration of tissue, mg/ml.

### Estimation of Neurotransmitters

#### Preparation of Aqueous Phase

The brain was mixed with 5 ml HCl–butanol. This mixture was homogenized for 1 min and further centrifuged at 2000 rpm for 10 min. The resultant supernatant (1 ml) was separated in which heptane 2.5 ml and 0.31 ml of HCl (0.1 M) were added. This mixture was vigorously shaken and was again centrifuged under the same conditions for the isolation of both phases. The upper organic layer was discarded, and the lower aqueous phase was used for the estimation of neurotransmitters, that is, serotonin, dopamine, and noradrenaline (Rahman and Eswaraiah, 2008).

#### Estimation of Serotonin Level

O-phthaldialdehyde (OPT) 0.25 ml was added to 0.2 ml of the aqueous phase. This mixture was then boiled up to 100°C for almost 10 min. It was allowed to cool at room temperature. Absorbance was measured at 440 nm. The blank solution was HCl 0.25 ml without OPT (Hira et al., 2020).

#### Estimation of Dopamine and Noradrenaline Levels

HCl (0.4 M) 0.05 ml and EDTA (0.1 ml) were added to 0.2 ml of aqueous phase, and then for oxidation process, 0.1 ml of iodine solution (0.1 M in ethanol) was added to this mixture. Sodium sulfite (Na_2_SO_3_) 0.1 ml was added to stop the oxidation process, and after one and a half minute, acetic acid (0.1 ml) was added. This reaction mixture was heated up to 100^°^C for about 6 min, and it was allowed to cool at room temperature. Absorbance was measured at 350 nm wavelength for dopamine, whereas at 450 nm, for noradrenaline. A blank solution was prepared by reversing the order of addition of oxidation process reagents, that is, first, Na_2_SO_3_ was added and then iodine (Ali et al., 2021).

### Quantification of Inflammatory Biomarkers NFK-β, TNF-α, and COX-2 With ELISA

The standard methods given in the ELISA kits were followed for the quantification of inflammatory biomarkers NFK-β, TNF-α, and COX-2. ELISA kits by Glory Science Co., Ltd were used for NFK-β (catalog No. 10947) and COX-2 (catalog No. 11076), and the Abcam ELISA kit was used for TNF-α (catalog No. ab46087).

### Statistical Analysis

GraphPad Prism version 5.0 software was used for the statistical evaluation of results. Data are expressed as mean ± SEM. One-way ANOVA, followed by Dunnett’s multiple comparison test, and two-way ANOVA, followed by the Bonferroni posttest, were used to interpret results. *p* < 0.05 was considered as a level of significance.

## Results

### Total Flavonoid Contents

Flavonoids and phenolics compounds are the major natural compounds that possess diverse activities. Flavonoid contents were measured by using the following regression line equation: y = 0.1697x + 0.02231 (R^2^ = 0.9). Total flavonoid contents in the methanolic extract of *V. odorata* were 2.407 mg of rutin equivalent/g of sample.

### Total Phenolic Contents

Phenolic contents were measured by using following regression line equation; y = 0.6360x + 0.09263 (R^2^ = 0.9). Total phenolic contents in the methanolic extract of *V. odorata* were 1.224 mg of gallic acid equivalent/gm of the sample.

### Evaluation of *In Vitro* Antioxidant Activity by Free Radical Scavenging DPPH Assay

**FIGURE 1:**
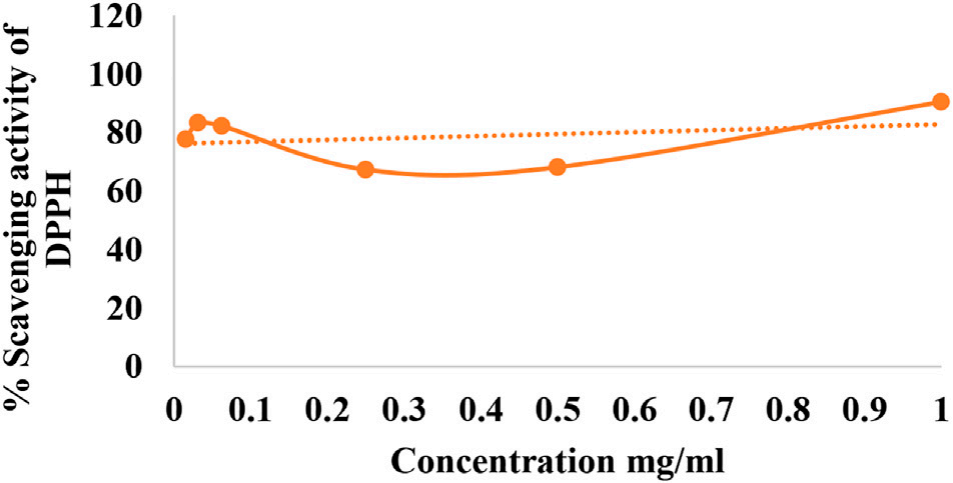
Percentage scavenging activity of DPPH.

The use of DPPH provides an easy and rapid way to evaluate antioxidant activity. The maximum percentage scavenging activity showed was at a concentration of 1 mg/ml, which was the maximum concentration used. Thus, percentage activity decreased with the reduction in the concentration of plant extract (Figure 1).

### Liquid Chromatography–Mass Spectrophotometry (GC-MS) Analysis

**FIGURE 2:**
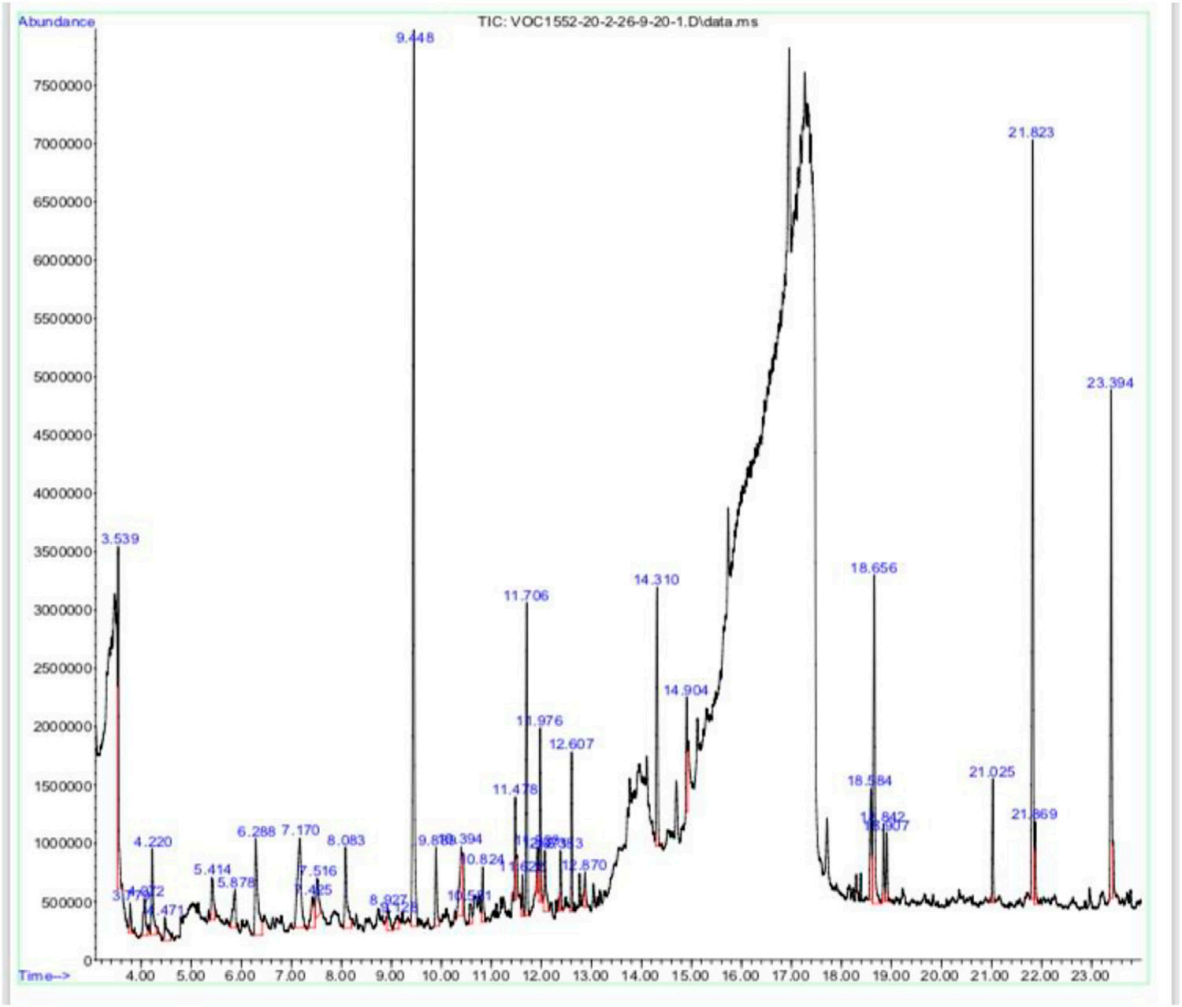
GC-MS analysis of *Viola odorata* methanolic extract.

Methanolic extract of plant *Viola odorata* was analyzed by GC-MS analysis, and various phytochemical constituents were detected like eugenol, benzene acetaldehyde, tetradecanoic acid, phytol, octadecatrenoic acid, undecane, gamma sitosterol, and bacchotricum (Table 1; Figure 2).

**TABLE 1:** Phytochemical constituent analysis of *Viola odorata* methanolic extract by using the GC-MS analysis

Sr.no	Retention time	Compound name	Molecular formula
1	3.60	Benzeneacetaldehyde	C_8_H_8_O
2	8.476	Eugenol	C_10_H_12_O_2_
3	11.721	2,5-Dihydroxy-4-isopropyl-2,4,6-cycloheptatrien-1-one	C_10_H_12_O_3_
4	11.841	Tetradecanoic acid	C_14_H_28_O_2_
5	12.616	3-Hydroxy-4,5-dimethoxybenzoic acid	C_9_H_10_O_5_
6	14.328	Card-20 (22)-enolide, 3-[(2,6-dideoxy-4-O-. beta.-D-glucopyranosyl-3-O-methyl-. beta.-D-ribo-hexopyranosyl) oxy]-5,14-dihydroxy-19-oxo	C_36_H_54_O_14_
7	13.373	Undecane, 3,8-dimethyl-	C_13_H_28_
8	18.611	Octadecatrenoic acid	C_18_H_30_O_2_
9	15.367	Phytol	C_20_H_40_O
10	21.038	Acetic acid n-octadecyl ester	C_20_H_40_O_2_
11	21.933	Di-isoctyl phthalate	C_24_H_38_O_4_
12	21.831	Glycerol 1-palmitate	C_19_H_38_O_4_
13	23.241	Bacchotricuneatin c	C_20_H_22_O_5_
14	23.681	Gamma-sitosterol	C_29_H_50_O
15	23.387	Octadecanoic acid	C_21_H_42_O_4_

### Behavioral Studies

#### Effect of *V. odorata* Methanolic Extract on Learning and Memory Using the Morris Water Maze Test

**FIGURE 3:**
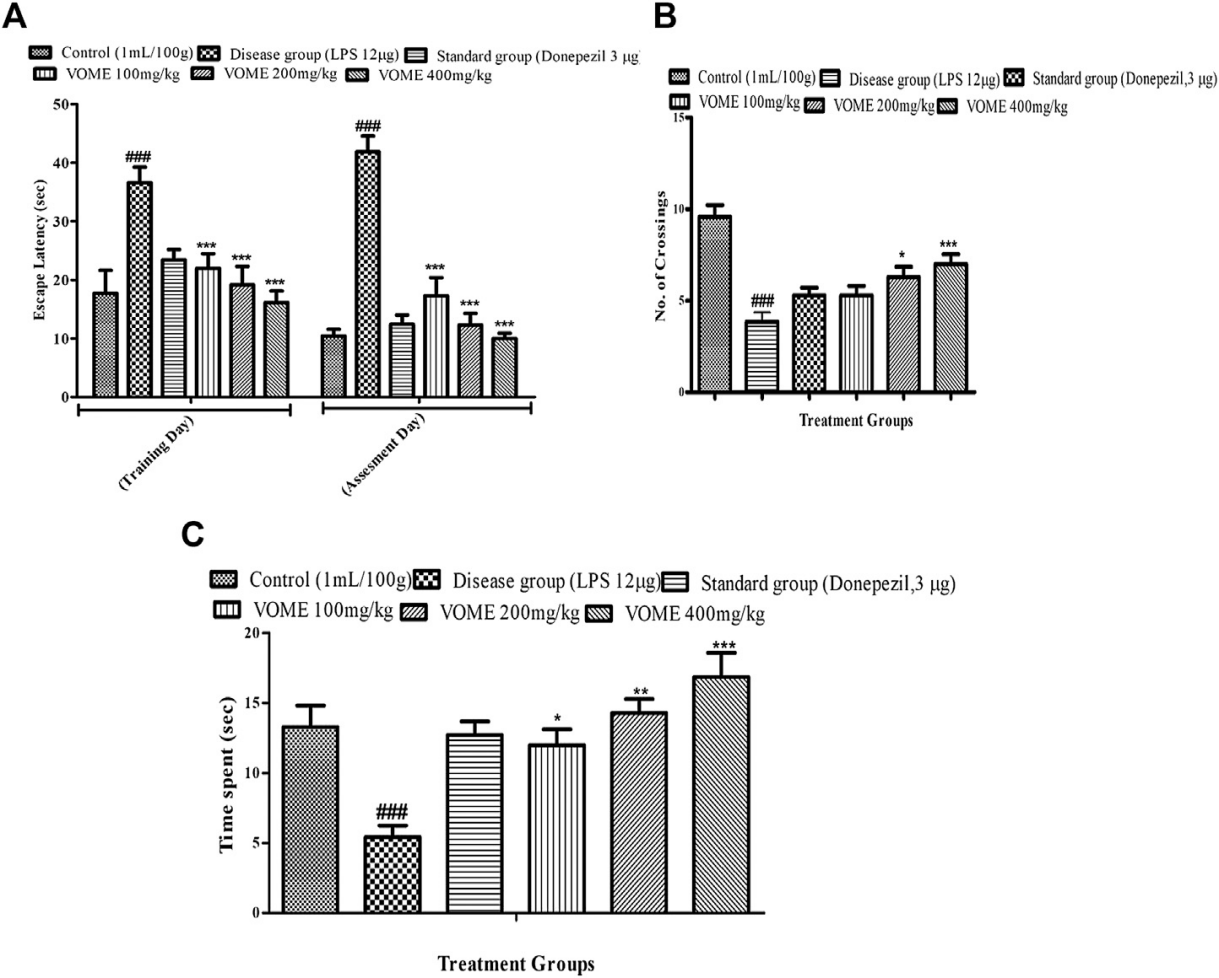
Effect of *V. odorata* methanolic extract on behavioral parameters of the Morris water maze test in mice. Histograms showing **(A)** escape latency, **(B**) No. of crossings, and **(C)** time spent in quadrant. Data are expressed as mean ± SEM (*n* = 10). *p < 0.05, **p < 0.01 and ***p < 0.001 as compared to the LPS treated group. ^###^ *p* < 0.001 as compared to the control group.

Spatial memory was assessed by observing the number of crossings of animals in the target quadrant (north) by finding the platform during the probe test. When LPS group was compared with the control group, a significant decrease in the number of crossing and time spent in the target quadrant were observed, while escape latency was also significantly increased in the LPS group. There is a significant (*p* < 0.001) decrease in the escape latency (EL) in the treatment groups as compared to the LPS group values on the training day and assessment day. If we compared EL on the training day with the assessment day values, then more decrease in EL was found on the assessment day (Figure 3A). Data showed in Figures 3B,C that the number of crossings and time spent in the target quadrant (north) were increased in all the treated groups, except the LPS group animals.

#### Effect of *V. odorata* Methanolic Extract in Cognitive Impairment Using the Elevated Plus Maze Test

**FIGURE 4:**
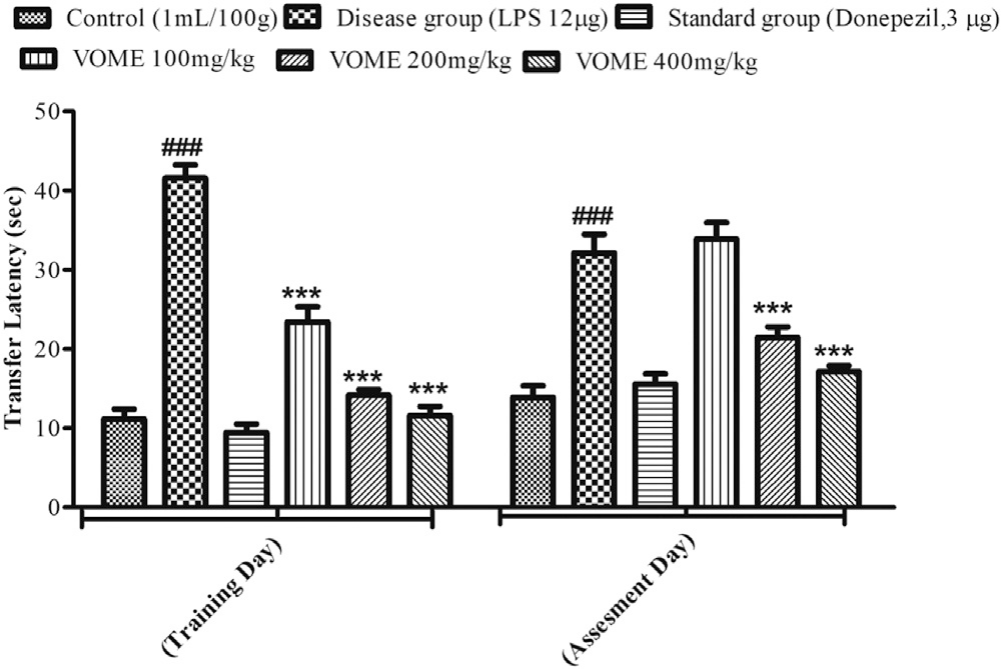
Histogram presenting effect of *Viola odorata* methanolic extract on transfer latency (sec) in the elevated plus maze test in mice. Values are expressed as mean ± SEM*, n =* 10. ***p < 0.001 when compared with the LPS-treated group*.* ^###^ *p* < 0.001 as compared to the control group.

The elevated plus maze test was performed on the 13th and 14th days of the study to assess the cognitive performance of mice. A significant rise in transfer latency (TL) was observed in the LPS group as compared with the control group. The TL was significantly (*p* < 0.001) decreased in a dose-dependent manner in the treatment groups on the training day, whereas on the assessment day, the treatment groups at 200 and 400 mg/kg experienced a significant (*p* < 0.001) decrease in TL as compared to the LPS treated group value (Figure 4).

#### Effect of *V. odorata* Methanolic Extract on Fear Learning and Memory Using the Fear Conditioning Test

**FIGURE 5:**
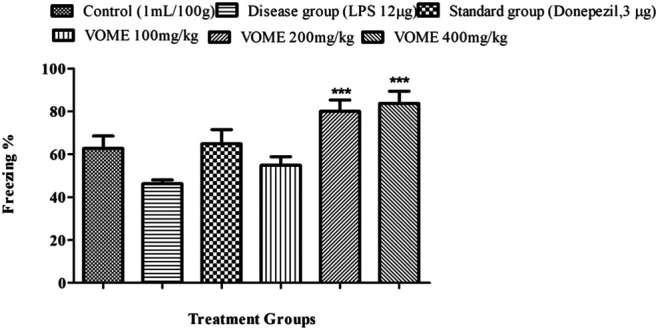
Effect of *Viola odorata* methanolic extract on freezing time (%) in the fear conditioning test in mice. Data are expressed as mean ± SEM, *n* = 10. ***p < 0.001, when compared with the LPS-treated group.

A fear conditioning test was performed on the 20th, 21st and 22nd days of study to assess the learning and memory of mice in fear conditions. Freezing time was calculated, which indicated the level of fear in mice. Percentage freezing response was increased significantly (*p* < 0.001) at 200 and 400 mg/kg VOME-treated groups with respect to the LPS group (Figure 5).

#### Effects of *V. odorata* Methanolic Extract on Memory Deficits Following the Passive Avoidance Test

**FIGURE 6:**
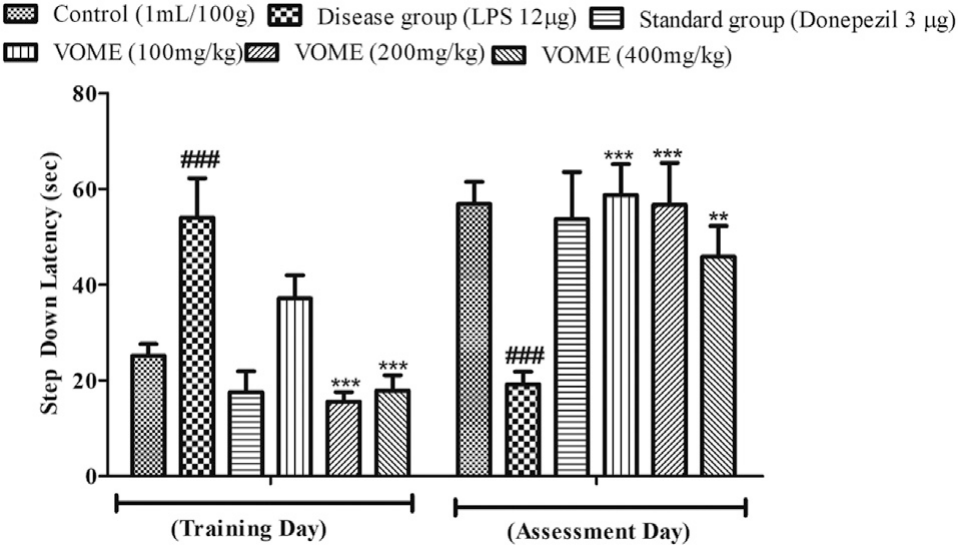
Histogram presenting the effect of *Viola odorata* methanolic extract on the step-down latency (sec) in the passive avoidance test in mice. Data are expressed as mean ± SEM, *n* = 10. ***p < 0.001 as compared to the LPS-treated group. ^###^ *p* < 0.001 as compared to the control group.

A passive avoidance test was performed to investigate episodic or long-term memory of mice by observing step-down latency (SDL). On the assessment day, significant reduction in the step-down latency was observed in the LPS group as compared to the control group. Results in Figure 6 indicated that SDL was increased significantly (*p* < 0.001) in the treatment groups on the assessment day as compared to the LPS group.

### Effects of *V. odorata* Methanolic Extract on Oxidative Stress Biomarkers

The oxidative damage was evaluated through estimation of biochemical marker (SOD, CAT, and GSH) levels in the brain tissue. SOD, CAT, GSH, and protein levels were significantly (*p* < 0.05) increased, whereas MDA levels were decreased in the treatment groups dose-dependently as compared to disease group values. AChE activity was significantly decreased in the treatment groups as compared to the disease group value (Table 2). SOD and protein contents were quantified using linear regression lines Y = 0.0095x × 0.1939 and Y = 0.0000751x × 0.0000746, respectively.

**TABLE 2:** Effects of *Viola odorata* methanolic extract on oxidative stress biomarkers and acetylcholinesterase

Treatment groups	Dose	SOD (µg/mg of brain tissue)	CAT (µg/mg of brain tissue)	Protein (µg/mg of brain tissue)	GSH (µg/mg of brain tissue)	MDA (µg/mg of brain tissue)	AChE (µmol/mg of brain tissue)
Control	1 ml/kg	22.2 ± 0.3	15.06 ± 0.005	1.789 ± 0.005	19.30 ± 0.1	1.88 ± 0.004	0.48 ± 0.005
Disease (LPS)	12 µg	13.0 ± 0.05^a^	6.91 ± 0.01^a^	0.406 ± 0.005^a^	16.65 ± 0.05^a^	1.924 ± 0.001^a^	1.245 ± 0.05^a^
Standard (donepezil)	3 µg	39.70 ± 0.25^b^	13.40 ± 0.005^b^	1.681 ± 0.005^b^	19.45 ± 0.05^b^	1.58 ± 0.0005^b^	0.765 ± 0.005^b^
VOME	100 mg/kg	14.0 ± 0.35^b^	12.16 ± 0.005^b^	0.685 ± 0.001^b^	15.55 ± 0.05^b^	1.38 ± 0.0005^b^	1.31 ± 0.005^b^
	200 mg/kg	19.0 ± 0.05^b^	16.46 ± 0.005^b^	0.934 ± 0.001^b^	17.25 ± 0.05^b^	1.15 ± 0.005^b^	0.985 ± 0.005^b^
	400 mg/kg	23.0 ± 0.45^b^	18.90 ± 0.001^b^	1.387 ± 0.005^b^	23.05 ± 0.05^b^	0.89 ± 0.0005^b^	0.435 ± 0.005^b^

Values are expressed as mean ± SEM. (*n* = 10).

a *p* < 0.05 as compared to the control group.

b *p* < 0.05 vs. the disease group.

### Effect of *V. odorata* Methanolic Extract on Neurotransmitters

**FIGURE 7:**
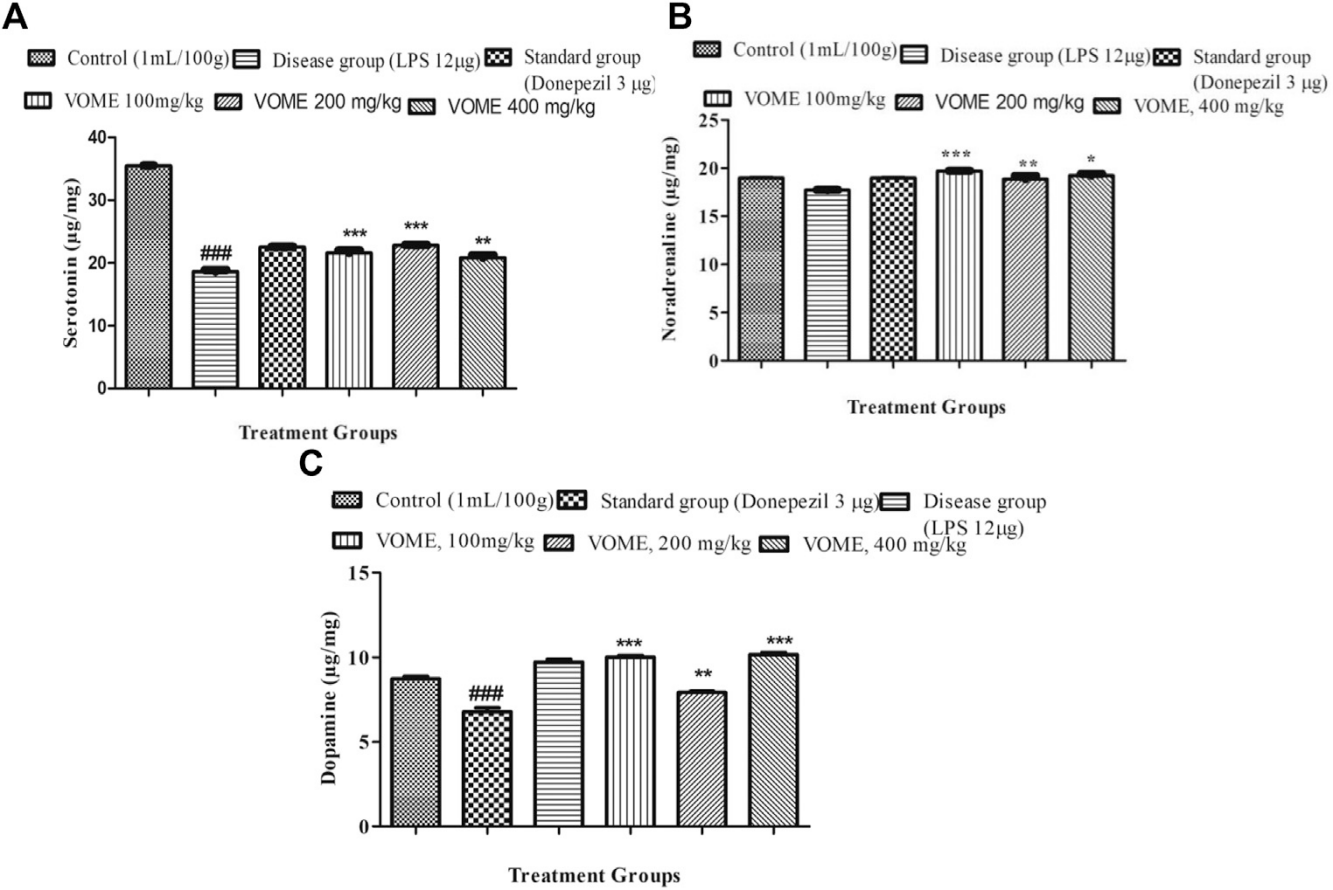
Effect of *Viola odorata* methanolic extract on neurotransmitters. **(A)** Histogram indicating results of the serotonin level in the mice brain. **(B)** Histogram indicating results of Noradrenaline level in the mice brain. **(C)** Histogram indicating results of dopamine level in the mice brain. Data are expressed as mean ± SEM, *n* = 10. *P<0.05, **p < 0.01 and ***p < 0.001 as compared to the LPS-treated group. ^###^ *p* < 0.001 as compared to the control group.

Serotonin, noradrenaline, and dopamine levels were quantified in the brain tissue using linear regression lines Y = 0.03144x × 01067; Y = 0.1008x × 0.2508; and Y = 0.2331x × 0.0164, respectively. The level of serotonin was significantly decreased in the brain of LPS treated animals; however, no difference was observed in the levels of noradrenalin and dopamine. There was a significant dose-dependent increase in the serotonin, noradrenaline, and dopamine (Figures 7A–C).

### Quantification of Inflammatory Biomarkers NFK-β, TNF-α, and COX-2 With ELISA

**FIGURE 8:**
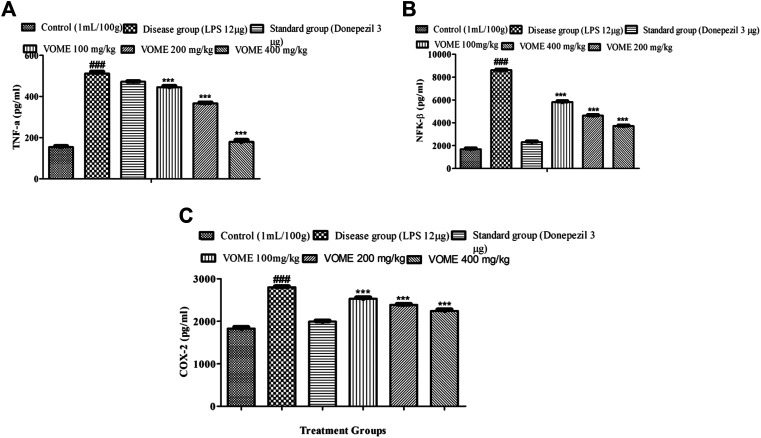
Effect of *Viola odorata* methanolic extract on inflammatory biomarkers. **(A)** Histogram presenting results of TNF-α in the mice brain. **(B)** Histogram presenting results of NFK-β in the mice brain. **(C)** Histogram presenting results of COX-2 in the mice brain. Data are expressed as mean ± SEM, *n* = 10. ***p < 0.001 as compared to the LPS-treated group. ^###^ *p* < 0.001 as compared to the control group.

Memory impairment is due to neurodegeneration and inflammation that occurs in the brain. Inflammatory biomarkers NFK-β, TNF-α, and COX-2 were raised in the LPS disease group, whereas in the treatment groups, these inflammatory biomarkers were significantly (*p* < 0.001) decreased dose-dependently (Figures 8A–C), which is indicative of therapeutic effectiveness of the treatment. Linear regression lines y = 0.0003 (X) + 0.1509 for TNF-α, y = 0.0000829 (X) + 0.2934 for NFK-β, and y = 0.0003(x) + 0.1509 for COX-2 were used for the quantification of the respective inflammatory biomarkers.

## Discussion

Various behavioral and biochemical studies were performed to explore the anti-Alzheimer potential of VOME. In the present study, phytochemicals like eugenol, gamma-sisterol, and phytol were identified with GC-MS. These compounds possess several pharmacological properties including anti-inflammatory and antioxidant potentials (Duke, 1992; Govindharajalu, 2015; Karthikeyan et al., 2017; Markkas and Barboza et al., 2018). Neuroinflammation and oxidative stress which were induced with LPS are causes of Alzheimer in the study (Pretorius et al., 2018). LPS causes significant memory impairment after I.C.V. administration into the mice. Pro-inflammatory cytokines, that is, TNF-α, IL-1β, and NFK-β, were produced in the glial cells that initiate the cascade of inflammatory reactions with LPS. These cytokines cause the deposition of amyloid proteins. LPS-induced inflammation stimulates the apoptosis in the mitochondria through increased levels of proapoptotic proteins which is implicated in many neurodegenerative diseases. Neuronal cell death following the LPS administration may cause the activation of cyclooxygenase-2 (COX-2), which is a prominent factor involved in the pathogenesis of cognitive dysfunction. COX-2 is the leading cause of aggregation of amyloid beta precursor proteins (APPs). LPS administration activates the COX-2 pathway under the influence of IL-1, leading to the production of prostaglandins (PG), which strongly affect the functioning of neurons involved in the memory (Griffin et al., 2013). LPS-treated animals showed elevated levels of cycloxegenase-2 (COX-2) in a dose-dependent manner.

The treated animals were assessed for spatial learning, long-term, and episodic memory by using Morris water maze, elevated plus maze, passive avoidance, and fear conditioning, respectively. Results of these models clearly indicated the memory improvement in treated mice that had received LPS.

An aggravated inflammatory reaction could be the reason for the increased production of ROS and nitric oxide (NO, through NO synthase) that suggests that inflammation is interlinked with oxidative stress. These ROS are responsible for mitochondrial dysfunction causing reduced GSH levels, which is the only antioxidant present in the mitochondria to remove hydrogen peroxide (Hira et al., 2020). The oxidative damage was evaluated through estimation of oxidative marker (SOD, CAT, and GSH) levels in the brain tissue. SOD, CAT, and GSH levels were significantly increased in VOME-treated groups when compared with the animals that had received LPS. The antioxidant property of the VOME might be due to the presence of eugenol, gamma-sisterol, and phytol; the active compounds that possessed the antioxidant property (Ogata et al., 2000). The mitochondrial dysfunction may cause the decreased utilization of ATP and acetyl Co-A, which affect the memory impairment by impeding the cholinergic transmission (Hira et al., 2019). Acetylcholinesterase enzyme causes the breakdown of acetylcholine (ACh), which is the prominent factor to suppress the production of inflammatory mediators (Tyagi et al., 2010). The findings of the present study revealed the raised levels of AChE in the LPS-treated animals that had suggested the excessive inflammatory response. Donepezil, an FDA-approved drug, is used in AD, which halts the breakdown of acetylcholine and ultimately, increases the acetylcholine level and cholinergic transmission at synapses. This might further confirm the cognitive improvement due to increased concentration of ACh (Wilkinson et al., 2004). The amyloid hypothesis suggests that AChE is also responsible for the excessive deposition of APP playing a crucial role in the pathogenesis of AD (Anand and Singh, 2013). The memory-enhancing effect of the methanolic extract of *V. odorata* could be attributed to the presence of eugenol, gamma-sisterol, and phytol in the plant under study.

Neurotransmitters (dopamine, serotonin, and noradrenaline) levels are decreased in AD. Low levels of NA initially results in anxiety and lack of attention, which later on progresses to AD. Noradrenaline transmission is affected due to the degeneration in noradrenergic neurons (Chalermpalanupap et al., 2013). Serotonin is related to cognitive abilities by mediating cholinergic transmission through acetylcholine. During the early stage of AD, dwindled dopaminergic neuronal activity is also reported in the progression of memory loss (Reinikainen et al., 1990). The treatment groups challenged with LPS exhibited an increased level of all neurotransmitters (dopamine, serotonin, and noradrenaline) in comparison to the disease group.

## Conclusion

The outcome of the present study proposes that the methanolic extract of *V. odorata* may be therapeutically effective in memory improvement by increasing oxidative stress biomarkers and neurotransmitters and decreasing pro-inflammatory markers. However, the exact mechanism by which it ameliorates memory functions is still unknown and needs further study at the molecular level.

## Data Availability

The original contributions presented in the study are included in the article/Supplementary Material, further inquiries can be directed to the corresponding authors.
